# Performance of creatinine-based equations for estimating glomerular filtration rate compared to endogenous creatinine clearance

**DOI:** 10.1590/2175-8239-JBN-2021-0109

**Published:** 2021-12-06

**Authors:** Gisele da Silva da Fonseca, Vandréa Carla de Souza, Sarah Assoni Bilibio, Vanessa Carobin, Lígia Facin, Ketelly Koch, Morgana Machado, Laurence Dubourg, Luciano da Silva Selistre

**Affiliations:** 1 Universidade de Caxias do Sul, Programa de Pós-Graduação em Ciências da Saúde Brasil, Caxias do Sul, RS, Brasil.; 2 Universidade de Caxias do Sul, Caxias do Sul, RS, Brasil.; 3 Hospital Geral de Caxias do Sul, Caxias do Sul, RS, Brasil.; 4 Université Claude Bernard Lyon 1, Lyon, França.

**Keywords:** Glomerular Filtration Rate, Creatinine, Regression Analysis, Renal Insufficiency, Chronic, Taxa de Filtração Glomerular, Creatinina, Análise de Regressão, Insuficiência Renal Crônica

## Abstract

**Introduction:**

The guidelines recommend estimating the glomerular filtration rate using serum creatinine-based equations as a predictor of kidney disease, preferably adjusted for local population groups.

**Methods:**

Cross-sectional study that evaluated the performance of four equations used for estimating GFR compared to endogenous creatinine clearance (ClCr) in 1,281 participants. Modification of Diet equations in Renal Disease Study Group (MDRD), Chronic Kidney Disease Epidemiology Collaboration (CKD-EPI), CKD-EPI with adjustment for local population (CKD-EPI local) and Full Age Spectrum (FAS) in comparison with endogenous creatinine clearance (ClCr). We used the Quantile Regression to calculate the median bias, interquartile range (IQR), Bland-Altman agreement analysis and 30% margin of error (P_30_).

**Results:**

The mean age of participants was 52.5 ± 16.5 years with 466 women (38%), median ClCr[IQR] of 92.0 [58.0; 122.0] mL/min/1.73 m^2^, with 320 (25%) participants presenting ClCr < 60 mL/min/1.73 m^2^. The performance of the local CKD-EPI and FAS equations were superior to MDRD and CKD-EPI in relation to variability (0.92 [0.89; 0.94]) and P_30_ (90.5% [88.7; 92, 0]). In the group with ClCr < 60 mL/min/1.73 m^2^, the local CKD-EPI and FAS equations showed less variability than the CKD-EPI and MDRD (0.90 [0.86; 0.98] and 1.05 [0.97; 1.09] vs. 0.63 [0.61; 0.68] and 0.65 [0.62; 0.70], P < 0.01) and best P_30_ (85.5) % [81.0; 90.0], 88.0% [84.0; 92.0] vs. 52.0% (46.0; 58.0) and 53.0% [47.0; 58 .5], P < 0.01).

**Conclusion:**

Local CKD-EPI and FAS equations performed better than CKD-EPI and MDRD when compared to ClCr.

## Resumo

**Introdução:**

As diretrizes recomendam a estimativa da taxa de filtração glomerular pelo uso de equações baseadas em creatinina sérica como preditor de doença renal, preferencialmente ajustadas para grupos populacionais locais.

**Métodos:**

Estudo transversal que avaliou o desempenho de quatro equações para estimativa da TFG em comparação com a depuração de creatinina endógena (DCE) em 1.281 participantes. Foram avaliadas as equações *Modification of Diet in Renal Disease Study Group* (MDRD), *Chronic Kidney Disease Epidemiology Collaboration* (CKD-EPI), CKD-EPI com ajuste para a população local (CKD-EPI local) e *Full Age Spectrum* (FAS) em comparação com a depuração de creatinina endógena (DCE). Utilizamos a Regressão Quantílica para cálculo do viés mediano, intervalo interquartil (IQR), análise de concordância de Bland-Altman e margem de erro de 30% (P_30_).

**Resultados:**

A idade média dos participantes era de 52,5 ± 16,5 anos com 466 mulheres (38%), mediana da DCE [IQR] de 92,0 [58,0; 122,0] mL/min/1,73 m^2^, com 320 (25%) participantes apresentando DCE < 60 mL/min/1,73 m^2^. A performance das equações CKD-EPI local e FAS foram superiores às MDRD e CKD-EPI em relação à variabilidade (0,92 [0,89; 0,94]) e P_30_ (90,5% [88,7; 92,0]). No grupo com DCE < 60 mL/min/1,73 m^2^, as equações CKD-EPI local e FAS apresentaram menor variabilidade que as CKD-EPI e MDRD (0,90 [0,86; 0,98] e 1,05 [0,97; 1,09] *vs*. 0,63 [0,61; 0,68] e 0,65 [0,62; 0,70], P < 0,01) e melhores P_30_ (85,5% [81,0; 90,0], 88,0% [84,0; 92,0] *vs*. 52,0% (46,0; 58,0) e 53,0% [47,0; 58,5], P < 0,01).

**Conclusão:**

As equações CKD-EPI local e FAS tiveram desempenho superior às CKD-EPI e MDRD, quando comparadas a DCE.

## Introduction

Glomerular filtration rate (GFR) is the best indicator of kidney function and is of great importance in screening for chronic kidney disease (CKD), especially in risk groups such as diabetics, hypertensive patients or those with a family history of CKD.

Ideally, GFR should be determined by reference methods such as urinary insulin clearance or plasma clearance of Iohexol and Ithalamate. However, in clinical practice, these tests are expensive and inaccessible in most nephrology centers. In Brazil, it is common to use 24-hour urinary creatinine clearance (ClCr) to estimate GFR, despite its limitations, especially errors in urine collection. Therefore, it is recommended to check the reliability of the sample with the excretion of urinary creatinine, which is reasonably constant in healthy individuals, being 20-25 mg/kg weight/24 hours for men and 15-20 mg/kg weight/24 hours for women.

The most commonly used marker of renal function is serum creatinine (SCr), but it can be affected by several biological factors, such as muscle metabolism, tubular secretion and laboratory dosage method. To minimize these variations, CKD management guidelines recommend the use of SCr-based mathematical equations as a non-invasive method to estimate GFR (eGFR)^1^. Recommended equations for adults are the Modification of Diet in Renal Disease Study Group (MDRD); and the Chronic Kidney Disease Epidemiology Collaboration (CKD-EPI)^2^^,^ 3. Both use SCr, gender, age and ethnicity (African-American or not) to calculate eGFR (Table 1). Another recently described equation is the Full Age Spectrum (FAS), based on the concept of median SCr normalized for the local population^4^.

**Table 1:** Equations used to estimate the Glomerular Filtration Rate

MDRD*	$\mathrm{eGFR}=175\times {\left(\mathrm{SCr}\right)}^{-1.154}\times \left[0.742\;\mathrm{if}\;\mathrm{women}\right]\times \left[1.159\;\mathrm{if}\;{\mathrm{black}}^{*}\right]$
**CKD-EPI**	$\mathrm{Women};\;\mathrm{SCr}\leq ,\;\mathrm{eGFR}=144\times {\left[\frac{\mathrm{SCr}}{0.7}\right]}^{-0.329}\times {\left[0.993\right]}^{\mathrm{Age}}$
	$\mathrm{Women};\;\mathrm{SCr}>0.7,\;\mathrm{eGFR}=144\times {\left[\frac{\mathrm{SCr}}{0.7}\right]}^{-1.209}\times {\left[0.993\right]}^{\mathrm{Age}}$
	$\mathrm{Men};\;\mathrm{SCr}\leq 0.9,\;\mathrm{GFR}=141\times {\left[\frac{\mathrm{SCr}}{0.9}\right]}^{-0.411}\times {\left[0993\right]}^{\mathrm{Age}}\times \left[1.159\;\mathrm{if}\;{\mathrm{black}}^{*}\right]$
	$\mathrm{Men};\;\mathrm{SCr}>0.9,\;\mathrm{eGFR}=141\times {\left[\frac{\mathrm{SCr}}{0.9}\right]}^{-1.209}\times {\left[0.993\right]}^{\mathrm{Age}}\times \left[1.159\;\mathrm{if}\;{\mathrm{black}}^{*}\right]$
**CKD-EPI local**	$\mathrm{Women};\;\mathrm{SCr}\leq 0.8,\;\mathrm{eGFR}=144\times {\left[\frac{\mathrm{SCr}}{0.8}\right]}^{-0.329}\times {\left[0.993\right]}^{\mathrm{Age}}$
	$\mathrm{Women};\;\mathrm{SCr}>0.8,\;\mathrm{eGFR}=144\times \left[\frac{\mathrm{SCr}}{0.8}\right]-1.209\times {\left[0.993\right]}^{\mathrm{Age}}$
	$\mathrm{Men};\;\mathrm{SCr}\leq 1.0,\;\mathrm{GFR}=141\times {\left[\frac{\mathrm{SCR}}{1.0}\right]}^{-0.411}\times {\left[0.993\right]}^{\mathrm{Age}}\times \left[1.159\;\mathrm{if}\;{\mathrm{black}}^{*}\right]$
	$\mathrm{Men};\;\mathrm{SCr}>1.0,\;\mathrm{eGFR}=141\times \left[\frac{\mathrm{SCr}}{1.0}\right]-1.209\times {\left[0.993\right]}^{\mathrm{Age}}\times \left[1.159\;\mathrm{if}\;{\mathrm{black}}^{*}\right]$
**FAS**	$\mathrm{Age}\leq 40\;\mathrm{years}:\;\mathrm{eGFR}=107.3\times \frac{\mathrm{SCr}}{1.0}$
	$\mathrm{Age}>40\;\mathrm{years}:\;\mathrm{eGFR}=107.3\times \frac{\mathrm{SCr}}{1.0}\times 0.988{\;}^{\mathrm{Age}-40}$
	$Q=1.0\frac{\mathrm{mg}}{\mathrm{dL}}\mathrm{inMen}\;\mathrm{and}\frac{0.8\mathrm{mg}}{\mathrm{dL}}\mathrm{in}\;\mathrm{Women}$

SCr: serum creatinine; CKD-EPI: *Chronic Kidney Disease–Epidemiology Collaboration*; MDRD: *Modification of Diet in Renal Disease Study*; FAS: *Full Age Spectrum*.

The present study evaluated the performance of four equations for estimating GFR: MDRD, CKD-EPI, CKD-EPI with adjustment for the local population (local CKD-EPI) and FAS using ClCr as reference standard, in adults from the northeast of Rio Grande do Sul.

## Methods

### Study population

Cross-sectional study that evaluated 2,427 adult individuals undergoing ClCr from January 1, 2010 to December 31, 2018. We excluded pregnant women and individuals with an inadequate 24-hour urinary sample. Data such as sex, age, weight, height, serum and urinary creatinine (UCr) and ClCr were extracted from the laboratory database, omitting the participant's name. All procedures were in accordance with the Brazilian regulatory standard 466/2012, and were approved by the institution's research ethics committee (CAAE 08129019.9.0000.5341).

### Laboratory evaluations

#### Endogenous creatinine clearance

The standard ClCr test was performed by measuring UCr in a urine sample collected within 24 hours and SCr in a blood sample on the same date. To check the integrity of the 24-hour urinary sample, we used an equation based on the creatinine excretion rate: [% = 100 (UCr in 24-hr, mg)/24 (weight, kg)] for men and [% = 100 (UCr in 24-hr, mg)/21 (weight, kg)] for women. The samples that did not reach the value of 60% to 140% were excluded^5^^,^ 6.

#### Creatinine dosage

The laboratory determination of SCr and UCr were obtained by the alkaline picrate method, Jaffé reaction traceable to the IDMS (isotope dilution mass spectrometry) method. The median SCr value, necessary to apply the FAS equation, was obtained based on 65,535 SCr measurements from a healthy adult population (18 to 90 years), from the same laboratory, in the period from January 2014 to December 2018. We obtained a median SCr of 1.0 mg/dL for men and 0.80 mg/dL for women.

#### Glomerular filtration rate estimation

The GFR was estimated with the four equations: MDRD, CKD-EPI, local CKD-EPI and FAS (Table 1) and we compared its performance with the ClCr as a reference standard in the general population and in individuals with ClCr <60 mL/min/1.73 m^2^.

#### Statistical analysis

The categorical variables were expressed as absolute and relative frequencies, and the numerical variables as median and interquartile range (IQR).

For the performance analysis between each equation and the ClCr, we used the following tools: 1) the median eGFR/ClCr ratio, to express the bias. The reason was chosen over the difference, to correct the heteroscedasticity of the data; 2) the interquartile range of the median ratio, to express its dispersion around the ratio; 3) Bland-Altman graph, with 95% limits of agreement (LoA); 4) Spearman's coefficient, evaluating the agreement between eGFR and ClCr; 5) 30% margin of error (P_30_), proposed by the KDOQI guideline, defined as the proportion of estimates (eGFR) that present results within the ClCr ± 30% range.

Data variability was estimated by the median ratio and the IQR. The P_30_ of each eGFR was evaluated comparing its results with the reference standard (ClCr), using the equation: (GEF/ClCr) x 100/ClCr. To calculate the median ratio, IQR and LoA, quantile regression was used.

The 95% confidence interval (95% CI) was calculated for all measurements through resampling (Bootstrapping) using the 2000 percentile technique.

All analyzes were performed using the R software for Windows version 4.0.2. A p value < 0.01 was considered for statistical significance.

## Results

### Characteristics of the population

**Figure 1:**
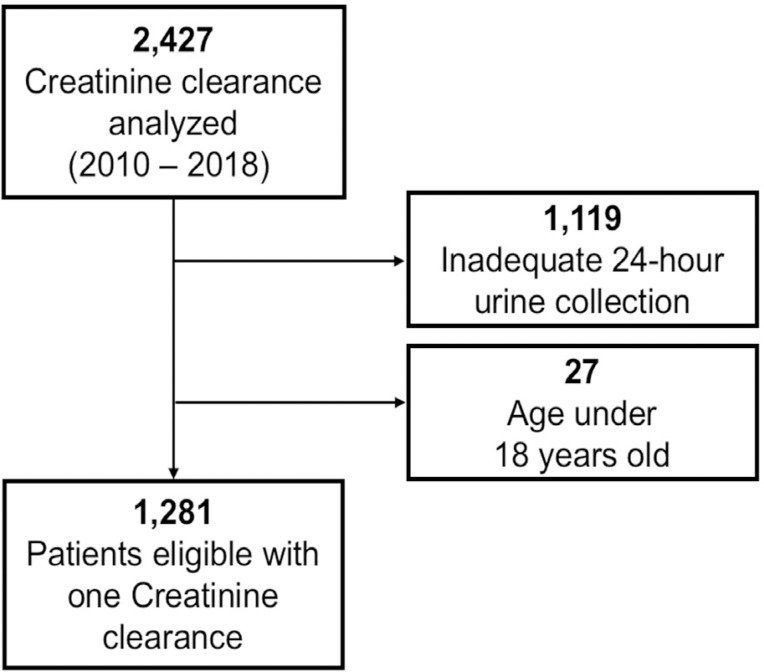
Participant selection flowchart.

During the study period, 2,427 ClCr results were obtained from individual participants, with 1,119 (46%) being excluded due to inadequate urinary collection and 27 (1%), under 18 years of age, with 1,281 being eligible for analysis (Figure 1).

The median age [IQR] of the participants was 53.0 [38.0; 65.0] years, 485 (38%) were female (Table 2). The median body mass index (BMI) [IQR] was 26.0 [24.0; 29.0] kg/m^2^, 255 (20.0%) classified as obese.

**Table 2:** Population Characteristics

Characteristics	Total population	ClCr < 60 mL/min/1.73 m^2^
Number of participants, n (%)	1.281 (100.0)	320 (25.5)
Mean age [IQR], years	53.0 [38.0; 65.0]	61.0 (49.0; 72.0)
≥ 65 years, n (%)	325 (26.5)	136 (42.5)
Females, n (%)	485 (38.0)	85 (25.5)
Median weight [IQR], Kg	74.0 [65.0; 85.0]	70.0 [65.0; 80.0]
Median height [IQR], cm	169 [162; 175]	170 [162; 175]
Median body surface [IQR], m^2^	1.84 [1.71; 1.98]	1.81 [1.70; 1.93]
Median BMI [IQR], Kg/m^2^	26.0 [24.0; 29.0]	25.0 [23.0; 28.0]
BMI ≥ 30.0, n (%)	255 (20.0)	56 (16.5)
Median serum creatinine [IQR], mg/dL	1.10 [0.80; 1.50]	2.90 [1.88; 4.30]
Median ClCr [IQR], mL/min/1.73 m^2^	94.0 [60.0; 124.0]	31.0 [21.0; 44.0]

ClCr: Endogenous Creatinine Clearance; IQR: Interquartile Range; BMI: body mass index

The median ClCr across the population [IQR] was 94.0 [60.0; 124.0] ml/min/1.73 m^2^. 320 participants (25.0%) were classified as CKD (< 60 mL/min/1.73 m2), with a median ClCr [IQR] of 31.0 [21.0; 44.0] mL/min/1.73 m^2^.

### Performance of equations

#### Variability

In the general population, the best median ratio was observed with the FAS equation, with a median eGFR/ClCr (95% CI) of 0.92 (0.89; 0.94) (Table 3, p < 0.01). In the CKD group, the FAS and CKD-EPI local equations exhibited less variability compared to the others (p < 0.01).

**Table 3:** Median ratio, IQR, limits of agreement, accuracy P_30_ and *Spearman* correlation for the eGFR equations

	Median ratio	IQR	LoA 2,5%	LoA 97,5%	P_30_ Accuracy	Spearman’s coefficient
	(CI 95%)	(CI 95%)	(CI 95%)	(CI 95%)	(CI 95%)	(CI 95%)
**Entire Population (N 1,281)**						
MDRD	0.74	0.18	0.47	0.98	50.5	0.895
	(0.51; 0.76)	(0.17; 0.19)	(0.44; 0.49)	(0.95; 1.02)	(45.0; 56.5)	(0.881; 0.915)
CKD-EPI	1.15	0.18	0.66	1.65	58.7	0.900
	(1.12; 1.17)	(0.17; 0.20)	(0.64; 0.69)	(1.62; 1.67)	(56.0; 61.6)	(0.881; 0.916)
CKD-EPI local	0.75	0.23	0.45	1.00	90.5	0.893
	(0.73; 0.77)	(0.22; 0.24)	(0.42; 0.47)	(0.98; 1.27)	(88.7; 92.0) ^‡^	(0.873; 0.910)
FAS	0.92	0.22	0.63	1.29	82.0	0.908
	(0.89; 0.94)*	(0.21; 0.23)	(0.60; 0.67)	(1.24; 1.33)	(79.7; 84.0)	(0.888; 0.922)
**Population with EEC < 60 mL/min/1.73 m^2^ (N = 320)**						
MDRD	0.65	0.20	0.37	0.86	53.0	0.880
	(0.62; 0.70)	(0.17; 0.22)	(0.28; 0.45)	(0.80; 1.04)	(47.0; 58.5)	(0.832; 0.916)
CKD-EPI	0.63	0.20	0.37	0.88	52.0	0.880
	(0.61; 0.68)	(0.18; 0.23)	(0.17; 0.40)	(0.86; 0.98)	(46.0; 58.0)	(0.830; 0.918)
CKD-EPI local	0.90	0.29	0.51	1.26	85.5	0.878
	(0.86; 0.98) ^‡^	(0.24; 0.32)	(0.24; 0.53)	(1.25; 1.50)	(81.0; 90.0) ^‡^	(0.827; 0.915)
FAS	1.05	0.24	0.60	1.40	88.0	0.862
	(0.97; 1.09) ^‡^	(0.20; 0.29)	(0.49; 0.68)	(1.39; 1.51)	(84.0; 92.0) ^‡^	(0.811; 0.901)

LoA: limits of agreement; P30: accuracy 30%; IQR: interquartile interval; CI 95%: 95% Confidence Interval

* P < 0.01 favoring FAS;

‡ P < 0.01 favoring CKD-EPI local

There was no significant difference in the precision assessed by the IQRs of the four equations, both in the general population and in the CKD (Table 3).

#### 30% margin of error

In the total population, the local CKD-EPI equation showed better P_30_ [CI 95%] than the other three equations: 90.5% [88.7; 92.0] (Table 3, p < 0.01). The P_30_ [CI 95%] of the MDRD, CKD-EPI and FAS equations were, respectively: 50.5% [45.0; 56.5], 58.7% [56.0; 61.6] and 82.0% [79.7; 84.0]. In the CKD group, the CKD-EPI local and FAS equations presented the best P_30_ [95% CI] than the other equations: 85.5% [81.0; 90.0] and 88.0% [84.0; 92.0] respectively (Table 3, p < 0.01).

#### Bland-altman concordance analysis

In the total population, in relation to the lower limit of agreement (LoA 2.5%) [CI 95%], the MDRD, CKD-EPI, local CKD-EPI, and FAS equations underestimated the ClCr 0.47 [0.44 ; 0.49], 0.66 [0.64; 0.69], 0.45 [0.42; 0.47] and 0.63 [0.60; 0.67], respectively). Regarding the upper limit of agreement (LoA 97.5%) [CI 95%], the CKD-EPI and FAS equations overestimated the ClCr: 1.65 [1.62; 1.67] and 1.29 [1.24; 1.33]), while the MDRD and local CKD-EPI equations showed a trend of agreement close to equality with the ClCr: 0.98 [0.95; 1.02] and 1.00 [0.98; 1.27], respectively (Table 3).

**Figure 2:**
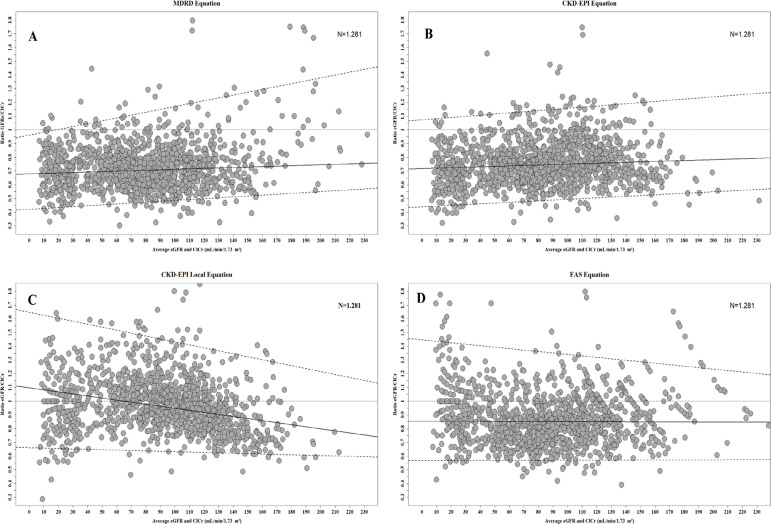
Bland-Altman plots showing the median GFRe / ClCr ratio versus the mean [(GFRe + ClCr) / 2] for each equation evaluated: MDRD (A), CKD-EPI (B), local CKD-EPI (C ) and FAS (D). The solid line represents the median ratio, the dotted lines represent the 95% limits of agreement.

In the CKD group, for LoA 2.5% [CI 95%], the MDRD, CKD-EPI, local CKD-EPI and FAS equations underestimated the ClCr: 0.37 [0.28; 0.45], 0.37 [0.17; 0.40], 0.51 [0.24; 0.53] and 0.60 [0.49; 0.68] (Table 3). Regarding the upper limit of agreement (LC 97.5%) [CI 95%], the MDRD and CKD-EPI equations underestimated the ClCr: 0.86 [0.80; 1.04] and 0.88 [0.86; 0.98] and the CKD-EPI local and FAS equations overestimated the ClCr: 1.26 [1.25; 1.50] and 1.40 [1.39; 1.51] (Table 3 and Figure 2).

**Figure 3:**
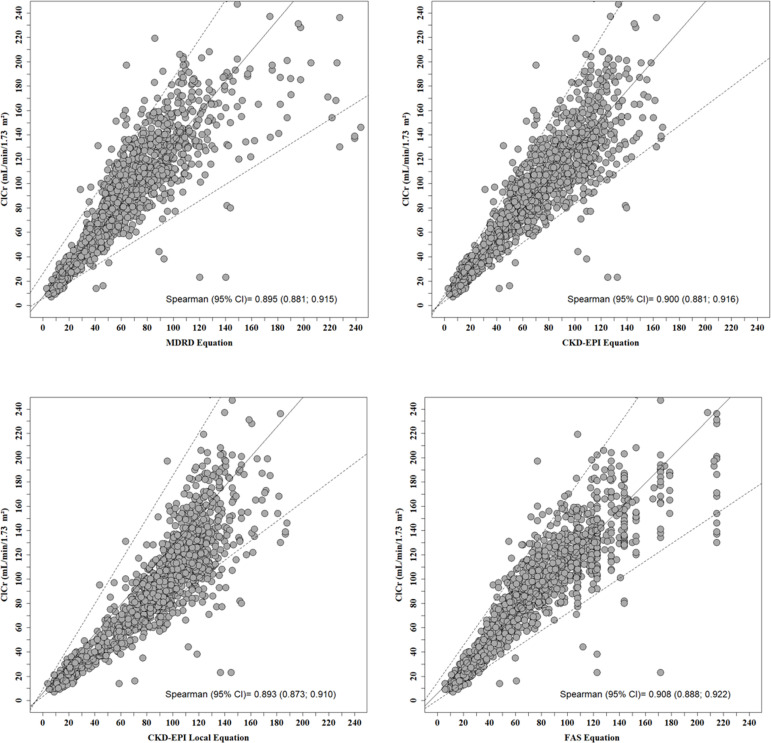
Quantile Regression Graph evaluating the correlation between MDRD (A), CKD-EPI (B), local CKD-EPI (C) and FAS (D) equations with ClCr. The solid line indicates the regression line and the dashed lines the 95% confidence interval.

The quantile regression graphs demonstrate good correlation between the ClCr and the MDRD, CKD-EPI, local CKD-EPI and FAS equations (Figure 3). In the Spearman's correlation, there were no significant differences between the equations and the ClCr (Table 3 and Figure 3), with robust correlation values in all assessments.

## Discussion

The present study evaluated the performance of four equations for estimating GFR compared to ClCr in a population of 1,281 adults, finding: 1) better accuracy of the local CKD-EPI equation; 2) satisfactory performance of the equation FAS; and 3) high probability of error (above 50%) in 24-hour urine collections for ClCr evaluation.

The local CKD-EPI had the best P_30_ among the four equations evaluated, with 90.5% of the estimated results within the range measured by the ClCr ± 30%, considered satisfactory for clinical interpretation as recommended by the KDIGO guideline^7^. The P_30_ of the CKD equation -Local EPI was superior to the equations recommended by the Society of Nephrology: MDRD and CKD-EPI. An equation for eGFR performs better when applied to populations similar to those in which it was developed, making it difficult for the same equation to work equally in different populations. The original CKD-EPI equation was developed in a North American population, with SCr modeled for mean values of 0.7 mg/dL for women and 0.9 mg/dL for men^3^. The authors recommend adjusting the SCr for local values^8^^-^ 10, as well as the KDIGO guideline^7^, however there are few studies that do. Following these guidelines, we adjusted the SCr for the population of the northeast region of Rio Grande do Sul, obtaining median values higher than those of North Americans, of 0.8 mg/dL for women and 1.0 mg/dL for men. The local adjustment of SCr led to a better performance of the CKD-EPI equation, demonstrating the importance of adjusting the equation model for each population evaluated, as suggested by the authors of the original CKD-EPI^3^. Other authors have already demonstrated an improvement in P_30_ for the populations. MDRD and CKD-EPI equations in relation to the original parameters when adjusting according to the characteristics of the local population^11^.

The FAS equation emerged as an alternative for evaluating eGFR due to its simplicity and adequate P30 in different age groups^4^^,^ 12. The concept of the FAS equation considers the decline in GFR only after 40 years based on physiological population studies with direct measurements of GFR^13^. However, the population that originated the FAS equation was exclusively European Caucasians, not being tested in other countries^12^. Our study is the first in Latin America to apply the FAS equation to a large sample of individuals and demonstrate its good bias performance and P_30_. Regarding P_30_, despite not having reached the recommended value - above 90% - in the total population, it presented a similar result in the CKD population (88.0 [84.0; 92.0]). The main advantage of the FAS equation is that it allows laboratories to make a relatively accurate estimate of GFR available to their clients, using only ClCr and gender as parameters and facilitating interpretation by the treating physician.

ClCr is widely used as a measure of renal function in clinical practice, but it tends to overestimate GFR^8^^,^ 14, mainly due to the proximal tubular secretion of 10% to 40% of urinary creatinine. Another relevant problem with ClCr is the high probability of error in urine collection within 24 hours^14^, despite the lack of quantitative data in the literature^15^^-^ 17. Our study found that 46% of the measurements had inadequate urine collection.

Among the strengths of this study are: 1) use of a representative sample of the population of the northeast region of Rio Grande do Sul; 2) use of SCr dosages standardized by the IDMS method; 3) use of robust statistical methods to evaluate the performance of equations. However, the study has some limitations that need to be listed. The retrospective character, based on a database, did not allow the evaluation of the ethnicity variable, a component of the MDRD and CKD-EPI equations, although there are reports in the literature that the GFR is independent of race or ethnicity^18^^-^ 20. It was also not possible to evaluation of morbidities, diets and treatments that could interfere with SCr. Furthermore, despite adjustments for local creatinine in FAS and local CKD-EPI, all equations studied here were validated in different populations, and may have a different performance than the original population. The small number of individuals with a GFR below 60 mL/min/1.73 m2 prevented the evaluation of the performance of the equations in CKD subgroups. Finally, the use of ClCr as a reference standard instead of a gold standard method for measuring GFR may have interfered with the interpretation of the results.

Finally, the present study reinforces the performance improvement of the equations that estimate the GFR after adjusting the SCr according to the characteristics of the population to be evaluated. In addition, it brings to light the large percentage of urinary sampling error for performing ClCr. It seems to us that an estimate of GFR with a correctly calibrated, standardized and adjusted SCr for the target population would have more reliable results and less cost when compared to ClCr.
